# Comparing Prognostic Scores and Inflammatory Markers in Predicting the Severity and Mortality of Acute Pancreatitis

**DOI:** 10.7759/cureus.39515

**Published:** 2023-05-26

**Authors:** Vasul Jain, Preetam Nath, Sudhir K Satpathy, Bandita Panda, Shubhransu Patro

**Affiliations:** 1 Department of General Medicine, Kalinga Institute of Medical Sciences, Bhubaneswar, IND; 2 Department of Gastroenterology and Hepatology, Kalinga Institute of Medical Sciences, Bhubaneswar, IND; 3 Department of Research and Development, Kalinga Institute of Medical Sciences, Bhubaneswar, IND

**Keywords:** lymphocyte/monocyte ratio, neutrophil/lymphocyte ratio, acute pancreatitis, red cell distribution, prognostic nutritional index

## Abstract

Background: Acute pancreatitis is an emergency gastrointestinal condition for which severity prediction is crucial during hospitalization. This study aimed to compare the diagnostic accuracy of inflammatory markers with gold standard scoring systems in predicting pancreatitis severity.

Materials and methods: A prospective, hospital-based, cohort study was conducted, including 249 patients diagnosed with acute pancreatitis via clinical examination. Laboratory investigations and radiological investigations were conducted. The diagnostic accuracy of the inflammatory markers neutrophil/lymphocyte ratio (NLR), lymphocyte/monocyte ratio (LMR), red cell distribution width (RDW), and prognostic nutritional index (PNI) was compared with gold standard prognostic scores, namely, the Acute Physiology and Chronic Health Evaluation II (APACHE II), Simplified Acute Physiology Score II (SAPS II), Bedside Index of Severity in Acute Pancreatitis (BISAP), and Systemic Inflammatory Response Syndrome (SIRS), in predicting primary and secondary outcomes. All values were analyzed using mean and standard deviation (SD). Sensitivity, specificity, positive predictive value, negative predictive value, and area under the receiver operating characteristic curve for mortality prediction were calculated for NLR, LMR, RDW, and PNI.

Results: Of 249 patients with acute pancreatitis (mean age: 39-43 years), 94 were classified as mild acute, 74 as moderately severe acute, and 81 as severe acute. The most common etiology was alcohol use (40.2%), followed by gallstones (29.7%), hypertriglyceridemia (6.4%), steroid use (4%), diabetic ketoacidosis (2.8%), hypercalcemia (2.8%), and complication of endoscopic retrograde cholangiopancreatography (2%). On day 1, mean values of NLR, LMR, RDW, and PNI were 8.23±5.11, 2.63±1.76, 15.93±3.64, and 32.84±8.13, respectively. Compared to APACHE II, SAPS II, BISAP, and SIRS on day 1, day 3, day 7, and day 14, the cutoff values for NLR were 4.06, 10.75, 8.75, and 13.75, respectively. Similarly, on day 1, the cutoff value of LMR was 1.95, and on day 1 and day 3, the cutoff values of RDW were 14.75% and 15%, respectively.

Conclusion: The results indicate that inflammatory biomarkers NLR, LMR, RDW, and PNI are comparable with gold standard scoring systems for predicting the severity and mortality of acute pancreatitis. NLR on day 7 was significantly associated with higher severity of illness. NLR on days 3, 7, and 14, LMR on day 1, and RDW on days 1 and 3 were significantly associated with mortality.

## Introduction

Acute pancreatitis is a frequent cause of gastrointestinal hospitalizations. Incidence has increased sharply in the last several decades to 20-40 per 100,000 people annually worldwide [1,2]. The mortality rate of acute pancreatitis is around 23% in the initial three days and increases to 53% by day 7, making proper management and prevention of complications essential. Numerous scoring systems such as the Acute Physiology and Chronic Health Evaluation II (APACHE II), Simplified Acute Physiology Score II (SAPS II), Bedside Index of Severity in Acute Pancreatitis (BISAP), Systemic Inflammatory Response Syndrome (SIRS), Modified Marshall, and GLASGOW are considered gold standards for predicting severity at admission in patients with acute pancreatitis. However, in rural areas with limited resources, these scoring systems are often too costly. Various clinical biomarkers (e.g., elevated levels of hematocrit, blood urea nitrogen (BUN), procalcitonin, and C-reactive protein (CRP)) have been used in the first 48 hours of hospitalization to aid in predicting illness severity [3-6]. Other cost-effective metrics include hematological parameters such as neutrophil/lymphocyte ratio (NLR), lymphocyte/monocyte ratio (LMR), red cell distribution width (RDW), and prognostic nutritional index (PNI) [7]. We thus aimed to compare the diagnostic accuracy of NLR, LMR, RDW, and PNI with the above gold standard prognostic scores in predicting severity and outcomes among patients with acute pancreatitis.

## Materials and methods

A hospital-based, prospective, cohort study was conducted at the Medicine and Gastroenterology Department in Pradyumna Bal Memorial Hospital, Kalinga Institute of Medical Sciences, Bhubaneswar. With approval from the ethics committee, the study recruited consecutive patients over age 18 who were admitted with acute pancreatitis, according to the 2012 Revised Atlanta Criteria, from December 2020 to June 2022. Patients were excluded if they had a history of oncological pathologies, hematological malignancies, anemia (iron deficiency, vitamin B12 deficiency, or chronic), thalassemia, or inflammatory bowel disease, or if they were pregnant. Patients were treated for acute pancreatitis according to standard guidelines and classified into different severity groups according to the Revised Atlanta Criteria (Table 1) [8].

**Table 1:** Classification of severity in acute pancreatitis as per the Revised Atlanta Criteria Source: [8]

Severity of acute pancreatitis	Local complications	Systemic complications
Mild	Absent	Absent
Moderate	Present	Transiently present
Severe	Present	Persistently present

After obtaining written consent, 249 patients were included. A detailed history, thorough examination, and laboratory testing were conducted, including complete blood count, serum electrolyte, serum amylase, serum lipase, serum hepatic and renal function, bilirubin, C-reactive protein, procalcitonin, and blood gas analyses. A radiological evaluation was performed using a transabdominal ultrasound and contrast-enhanced CT scan of the whole abdomen on days 5-7 of admission. APACHE II, SAPS II, BISAP, and SIRS scores were recorded in the first 24 hours. NLR, LMR, RDW, and PNI levels were calculated at the time of admission and on day 3, day 7, and day 14. Assessment of diagnostic accuracy included predicting acute pancreatitis as a primary cause of mortality and as a secondary cause of morbidity (duration of ICU and hospital stay) and the need for intervention (surgical, radiological, and endocrine). These parameters then were compared with the previously mentioned gold standard severity scores in terms of prognostic accuracy.

All qualitative parameters are presented in numbers and percentages. Quantitative parameters are presented as means, standard deviations, and medians with interquartile ranges, depending on the distribution. For all four scores, predictive diagnostic parameters including sensitivity, specificity, positive predictive value, negative predictive value, and area under the receiver operating characteristic curve were assessed for severity and mortality prediction. The Kruskal-Wallis test was applied for the hypothesis test of multiple variables (NLR, LMR, RDW, etc.) in severity prediction. A null hypothesis has been established in all independent variables. The Mann-Whitney U test was also applied to validate the null hypothesis. A p-value of less than 0.05 was considered significant. Data analysis was done using STATA software.

## Results

**Figure 1:**
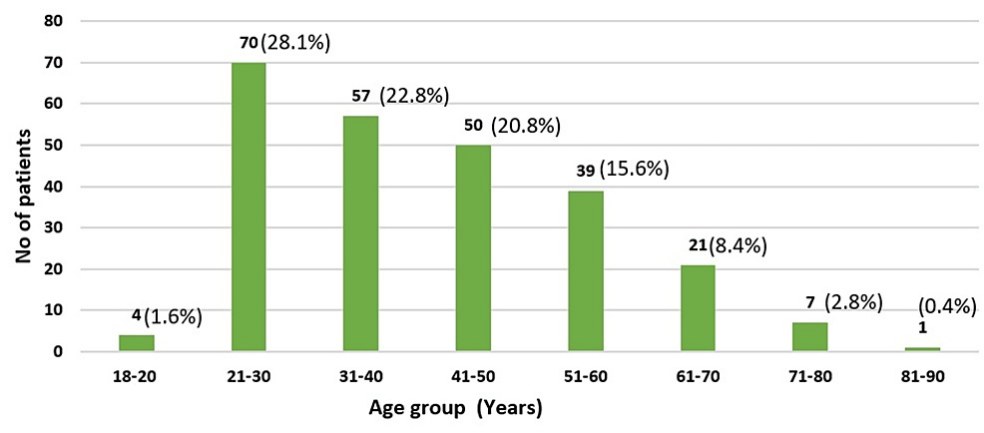
Age distribution in the study population of acute pancreatitis (N=249)

**Figure 2:**
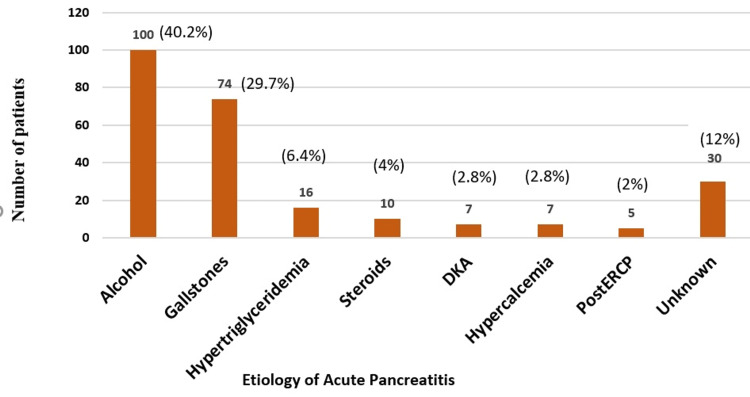
Etiology of acute pancreatitis in the total study population (N=249) DKA: diabetic ketoacidosis, ERCP: endoscopic retrograde cholangiopancreatography

Of 249 patients enrolled, 94 (37.8%) were diagnosed with mild acute pancreatitis, 74 (29.7%) with moderately severe acute pancreatitis, and 81 (32.5%) with severe acute pancreatitis. The mean age was 39±14.4 to 43.4±16.02 years, with most (28.10%) aged 21-30, 15.66% aged 51-60, 8.43% aged 61-70, and 2.81% aged 71-80 (Table 2 and Figure 1). Most (70.2%) were male, and 29.8% were female. Regarding comorbidities, 58 (23.2%) had diabetes mellitus, 18 (7.2%) had hypertension, six (2.4%) had chronic kidney disease, and two (0.8%) had coronary artery disease. Many (106, 42.5%) consumed alcohol, and 87 (34.9%) had SIRS. Alcohol consumption (40.2%) was the most common etiology, followed by gallstones (29.7%), hypertriglyceridemia (6.4%), steroid use (4%), diabetic ketoacidosis (2.8%), hypercalcemia (2.8%), post-endoscopic retrograde cholangiopancreatography (2%), and unknown cause (12%). Figure 2 summarizes the results.

**Table 2:** Clinico-demographic features of mild, moderate, and severe acute pancreatitis *Significant SD: standard deviation, ICU: intensive care unit, NLR: neutrophil/lymphocyte ratio, LMR: lymphocyte/monocyte ratio, RDW: red cell distribution width, BISAP: Bedside Index of Severity in Acute Pancreatitis, APACHE II: Acute Physiology and Chronic Health Evaluation II, SAP II: Simplified Acute Physiology Score II

Parameters	Mild	Moderate	Severe	Value
Age (years) (mean±SD)	43.4±16.02	39±14.4	41.43±13.35	0.161
Gender				
Male	29.3%	18.47%	22.8%	0.091
Female	8.43%	11.2%	9.63%	
Comorbidities				
Diabetes mellitus	9.63%	7.22%	6.42%	0.645
Hypertension	2%	2%	3.2%	0.501
Chronic kidney disease	0.4%	11.2%	9.63%	0.326
Coronary artery disease	0	0.8%	0	-
Etiology				
Alcohol use	18.8%	12.8%	10.8%	0.040*
Incidence of SIRS	11.2%	11.2%	12.44%	0.398
Length of hospital stay in days	9.78±5.74	10.38±5.69	11.79±8.05	0.05*
Length of ICU stay in days	-	6.33±4.93	7.39±6.07	0.001*
Number of organ failure	37.7%	15.2%	12.04%	0.001*
Inflammatory markers				
NLR	8.62±5.26	7.59±4.71	8.3±5.28	<0.05*
LMR	2.34±1.46	2.64±1.46	2.95±2.09	<0.05*
RDW (%)	15.54±3.43	16.37±3.7	15.9±3.8	<0.05*
Prognostic scores				
BISAP	0.48±0.63	0.43±0.74	1.22±1.37	<0.05*
APACHE II	5.55±3.63	5.5±3.35	7.51±5.25	<0.05*
SAPS II	21.26±11.51	18.96±7.45	25.07±11.53	<0.05*
Mortality	0%	0%	8.03%	

The average hospital stay was 10.61±6.6 days, as observed in 36.5% of patients. For 26.5% of patients, hospital stay exceeded 14 days. Only 4.4% of patients were discharged by day 3. ICU admission was required in 14.5% of cases. Multiple organ failure was observed in 8% of patients, compared to 26.9% of patients with single organ failure. In total, 8% of patients died while admitted (Table 2).

**Figure 3:**
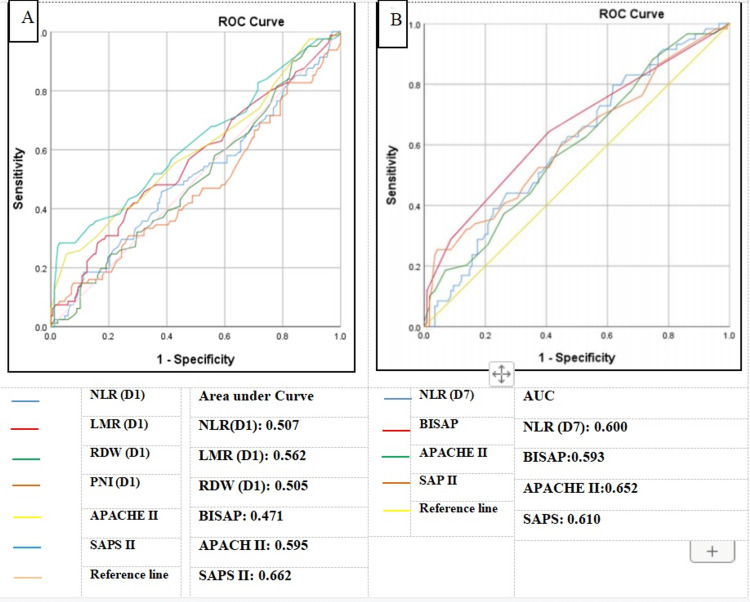
Assessment of inflammatory markers (NLR, LMR, and RDW) in comparison to the gold standard scoring systems (BISAP, APACHE II, and SAPS II) for the prediction of disease severity and mortality A: ROC curve of NLR, LMR, and RDW on day 1 in comparison with BISAP, APACHE II, and SAPS II for disease severity prediction B: ROC curve of NLR on day 7 in comparison with BISAP, APACHE II, and SAPS II for disease severity prediction NLR: neutrophil/lymphocyte ratio, LMR: lymphocyte/monocyte ratio, RDW: red cell distribution width, PNI: prognostic nutritional index, BISAP: Bedside Index of Severity in Acute Pancreatitis, APACHE II: Acute Physiology and Chronic Health Evaluation II, SAP II: Simplified Acute Physiology Score II, ROC: receiver operating characteristic

Inflammatory markers NLR, LMR, RDW, and PNI with interquartile ranges (IQRs) were analyzed for all patients, with median values of 7.4% (IQR=6.55), 2.1% (IQR=1.7), 14.9% (IQR= 2.1), and 31.08% (IQR=8.64), respectively. Prognostic scores of acute pancreatitis were determined using APACHE II, BISAP, and SAPS II methods for all patients, with mean scores of 6.17±4.24, 0.71±1.02, and 21.82±10.72, respectively. Using receiver operating characteristic curve analysis, these values were compared with NLR, LMR, RDW, and PNI for sensitivity and specificity. On day 7, the cutoff value of NLR was 4.06 (p<0.05) with a sensitivity of 79.7% and specificity of 38.3% in predicting disease severity (Figure 3A, 3B).

**Figure 4:**
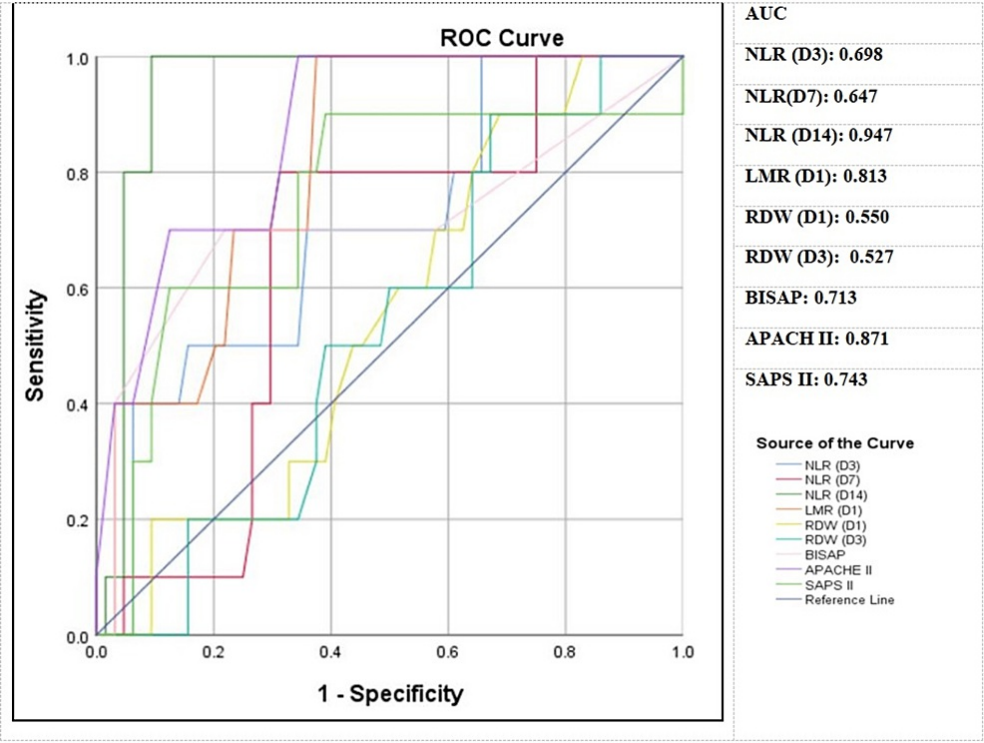
Assessment of inflammatory markers (NLR, LMR, and RDW) in comparison to the gold standard scoring systems (BISAP, APACHE II, and SAPS II) for mortality prediction ROC curve of NLR (day 3, day 7, and day 14), LMR (day 1), and RDW (day 1 and day 3) in comparison with BISAP, APACHE II, and SAPS II for mortality prediction NLR: neutrophil/lymphocyte ratio, LMR: lymphocyte/monocyte ratio, RDW: red cell distribution width, BISAP: Bedside Index of Severity in Acute Pancreatitis, APACHE II: Acute Physiology and Chronic Health Evaluation II, SAP II: Simplified Acute Physiology Score II, ROC: receiver operating characteristic, AUC: area under the curve, D: day

Accuracy indexes in a particular area under the curve revealed that on day 3, the cutoff value of NLR was 10.75 (p<0.05) with 70% sensitivity and 64.1% specificity for mortality prediction. Similarly, on day 7 and day 14, NLR had cutoff values of 8.75 and 13.75 (p<0.05), respectively, with sensitivity rates of 80% and 100% and specificities of 68.8% and 90.6%. On day 1, the cutoff value of LMR was 1.95 (p<0.05) with 100% sensitivity and 62.5% specificity. The receiver operating characteristic analysis of RDW revealed that on days 1 and 3, the cutoff value was 14.75 (p<0.05) with a sensitivity of 90% and specificity of 31.3% and 32.3%, respectively (Figure 4).

## Discussion

The diagnostic accuracy of inflammatory markers NLR, LMR, RDW, and PNI for predicting severity and mortality in 249 patients with acute pancreatitis was compared to gold standard prognostic scores. Clinico-demographic features were recorded. The mean age was 41.44±14.78 years, in which mostly male (>50%) patients participated, similar to studies by Li et al. [7] and Zhou et al. [9]. As far as illness severity, 32.5% of patients had severe cases, which is consistent with the observations of Zhou et al. [9]. The most common comorbidity was diabetes mellitus (23.2%), followed by hypertension (7.2%), chronic kidney disease (2.4%), and coronary artery disease (0.8%). A significant difference was not observed among severity levels, consistent with the findings of Zhou et al. [9].

SIRS was observed in 34.9% of patients in our study, consistent with the findings of Singh et al. [10], who concluded that SIRS is more reliable than APACHE II for predicting the severity of acute pancreatitis. Compared to patients with mild acute pancreatitis in this study, patients with severe acute pancreatitis had higher incidences of SIRS; higher rates of organ failure; longer hospital stays; higher mean NLR, LMR, RDW, BISAP, SAPS II, and APACHE II values; and higher mortality.

Numerous scoring systems have been established in predicting outcome severity in acute pancreatitis. Scores such as BISAP and SIRS are less complicated than APACHE II, but multifactorial scoring methods are laborious and call for considerable measures. CT scans and levels of C-reactive protein, hematocrit, and procalcitonin have also been used to predict the severity of acute pancreatitis, with varying degrees of success. LMR and NLR, which are determined from differential white blood cell counts, can indicate the magnitude of the inflammatory process in a particular disease condition. RDW, which measures the volume of red blood cell fluctuation, is also linked to inflammatory processes and is used to assess anemia and predict in-hospital mortality in patients with sepsis [11].

The mean values of NLR, LMR, RDW, and PNI on day 1 among patients in this study were 8.23±5.11, 2.63±1.76, 15.93±3.64, and 32.84±8.13, respectively. The mean BISAP, APACHE II, and SAPS II scores were 0.71±1.02, 6.17±4.24, and 21.82±10.7, respectively. Hospital stays exceeded seven days in 42.1% of cases. Li et al. [7] reported a mean NLR of 8.46 in patients with mild acute pancreatitis, which increased to 19.65 as the illness worsened to severe acute pancreatitis; LMR was 1.88 in patients with mild acute pancreatitis and decreased to 1.03 as the illness progressed to severe acute pancreatitis. Zhou et al. [9] found that the LMR value increases in severity in parallel with NLR, Ranson, sequential organ failure assessment, and BISAP values, along with significant differences between mild acute and severe acute pancreatitis.

The diagnostic accuracy of NLR was analyzed based on the receiver operating characteristic curve. The cutoff value of NLR on day 7 was 4.06 with 79.7% sensitivity and 38.3% specificity for severity prediction. Dancu et al. [12] reported a day 1 cutoff value of NLR of 9.6 with 65% sensitivity and 70% specificity for acute pancreatitis severity; after 48 hours, it decreased to 6.15 with 100% sensitivity and 63% specificity. Zhou et al. [9] found a 10.31 cutoff value of NLR with 64.3% sensitivity and 77.1% specificity for 28-day mortality prediction. In our study, acute pancreatitis mortality on day 3 was associated with an NLR cutoff of 10.75 with 70% sensitivity and 64.1% specificity. Similarly, NLR on day 7 and day 14 had cutoff values of 8.75 and 13.75, sensitivities of 80% and 100%, and specificities of 68.8% and 90.6%, respectively, for mortality prediction.

Regarding predictions of mortality in our study, LMR on day 1 had a cutoff value of 1.95 with 100% sensitivity and 62.5% specificity; RDW on days 1 and 3 had cutoff values of 14.75% and 15% with sensitivities of 90% and 90% and specificities of 31.3% and 32.3%, respectively. Li et al. [7] found that at a 16.64 cutoff value, NLR was 82.4% sensitive and 75.6% specific in predicting mortality of acute pancreatitis, whereas LMR at a 1.4 cutoff value was 82.4% sensitive and 57.3% specific and RDW at a cutoff value of 13% was 94.1% sensitive and 54.3% specific. Zhou et al. [9] found that at a 12.20 cutoff value, NLR was 85.7% sensitive and 84.2% specific in predicting 28-day mortality in patients with acute pancreatitis and that RDW at a 13.55% cutoff value was 100% sensitive and 74.7% specific in predicting the severity of acute pancreatitis [13-15].

Comparing patients with mild acute pancreatitis to patients with severe acute pancreatitis in our study, significant differences (p<0.05) were observed in the incidence of SIRS (11.20% versus 12.44%), length of hospital stay (9.78±5.74 versus 11.79±8.05), length of ICU stay (0 versus 7.39±6.07), number of organ failures, NLR on day 7 (6.96±6.19 versus 7.96±5.11), BISAP score (0.48±0.63 versus 1.22±1.37), APACHE II score (5.55±3.63 versus 7.51±5.25), SAPS II score (21.26±11.51 versus 25.07±11.53), and rate of mortality (0% versus 8%), based on the results of chi-square and Student’s t-tests. Zhou et al. [9] similarly compared patients with severe versus mild acute pancreatitis and found significantly lower NLR, RDW, sequential organ failure assessment, BISAP, APACHE II, and Ranson scores among mild cases (p<0.05). Li et al. [7] observed that patients with mild acute pancreatitis had significantly lower NLR values, RDW values, and mortality rates, compared to patients with severe acute pancreatitis (p<0.05).

In our study, the incidence of SIRS, the severity of acute pancreatitis, hospital stay length (10.52±6.42 versus 11.7±8.46), ICU stay length, number of organ failures, NLR (8.12±5.11 versus 9.33±5.07), LMR level (2.53±1.63 versus 3.76±2.58), RDW measure (15.8±6.07 versus 15.5±2.18), BISAP score (0.56±0.86 versus 2.4±1.19), APACHE II score (5.45±3.41 versus 14.45±4.11), and SAPS II score (20.53±9.73 versus 36.55±10.73) were significantly lower among patients who were discharged compared to patients who died in the hospital. Zhou et al. [9] similarly observed that compared to patients who died during their study period, those who survived had significantly lower NLR levels, RDW levels, BISAP scores, sequential organ failure assessment scores, APACHE II scores, and Ranson scores (p<0.05). Li et al. [7] also observed that compared to non-survivors, patients who survived had significantly lower NLR levels but higher LMR and PNI levels (p<0.05) (Table 3).

**Table 3:** Prediction performance report of NLR, LMR, and RDW for severity and mortality in acute pancreatitis NLR: neutrophil/lymphocyte ratio, LMR: lymphocyte/monocyte ratio, RDW: red cell distribution width, MAP: mild acute pancreatitis, MSAP: moderately severe acute pancreatitis, SAP: severe acute pancreatitis, SOFA: sequential organ failure assessment, BISAP: Bedside Index of Severity in Acute Pancreatitis, PNI: prognostic nutritional index, CRP: C-reactive protein, BUN: blood urea nitrogen

Study	Year	Sample size	Etiology	MAP	MSAP	SAP	Mortality	Result
Zhou et al. [9]	January 2014 to December 2017	406	Biliary (52.2%)	237	113	56	14	RDW was superior to other laboratory predictors in the prediction of not only SAP but also the mortality of acute pancreatitis. The combination of SOFA and RDW or BISAP and RDW could improve the predicting performance of single SOFA, BISAP, or RDW.
Li et al. [7]	July 2013 to August 2015	359	Gallstones (50%)	197	76	86	31	NLR, PNI, CRP, and RDW were independently associated with overall survival.
Dancu et al. [12]	January 1, 2018, to June 2019	216	Biliary (57%)	124	67	25	19	48-hour NLR and 48-hour CRP were independently associated with SAP, and BUN had a good predictive performance.
Our study	December 2020 to June 2022	249	Alcohol (40.2%)	94	74	81	20	NLR on day 7 was associated with severity and NLR on days 3, 7, and 14, LMR on day 1, and RDW on days 1 and 3 were associated with mortality in acute pancreatitis.

The limitation of the study was the low sample size during the study period.

## Conclusions

Early aggressive management and resuscitation are vital in treating acute pancreatitis, so severity prediction during hospitalization is important. Numerous scoring methods have been used for severity prediction, but they are impractical in many settings due to cost and resource limitations. Inflammatory indicators such as NLR, LMR, and RDW are easy and cost-effective methods and are comparable to multiparameter scoring systems for assessing severity and mortality risk prediction in patients with acute pancreatitis. NLR on days 1 and 7, and LMR and RDW on day 1 are comparable with APACHE II and SAP II for severity prediction. For mortality prediction, NLR on days 3 and 7, LMR on day 1, and RDW on days 1 and 3 are comparable with APACHE II, BISAP, and SAP II in acute pancreatitis.
